# Physical and emotional health outcomes after 12 months of public-sector antiretroviral treatment in the Free State Province of South Africa: a longitudinal study using structural equation modelling

**DOI:** 10.1186/1471-2458-9-103

**Published:** 2009-04-15

**Authors:** Edwin Wouters, Christo Heunis, Dingie van Rensburg, Herman Meulemans

**Affiliations:** 1 Department of Sociology and Research Centre for Longitudinal and Life Course Studies, University of Antwerp, Sint-Jacob Street 2, 2000 Antwerp, Belgium; 2 Centre for Health Systems Research & Development, University of the Free State, Nelson Mandela Avenue, Bloemfontein, South Africa

## Abstract

**Background:**

African and Asian cohort studies have demonstrated the clinical efficacy of antiretroviral treatment (ART) in resource-limited settings. However, reports of the long-term changes in the physical and emotional quality of life (QoL) of patients on ART in these settings are still scarce. In this study, we assessed the physical and emotional QoL after six and 12 months of ART of a sample of 268 patients enrolled in South Africa's public-sector ART programme. The study also tested the impact of the adverse effects of medication on patients' physical and emotional QoL.

**Methods:**

A stratified random sample of 268 patients undergoing ART was interviewed at baseline (< 6 months ART) and follow-up (< 12 months ART). A model of the relationships between the duration of ART, the adverse effects of medication, and physical and emotional QoL (measured using EUROQOL-5D) was tested using structural equation modelling.

**Results:**

The improved physical and emotional QoL shown at baseline was sustained over the 12-month study period, because treatment duration was not significantly associated with changes in the patients' QoL. Physical QoL significantly and positively influenced the patients' emotional QoL (subjective well-being [SWB]) (β = 0.33, *P *< 0.01). Longitudinal data showed that patients reported significantly fewer adverse effects at follow-up than at baseline (β = -0.38, *P *< 0.001) and that these adverse effects negatively influenced physical (β = -0.27, *P *< 0.01) and emotional QoL (β = -0.15, *P *< 0.05).

**Conclusion:**

This study provides evidence that the South African public-sector ART programme is effective in delivering sustained improvement in patient well-being. However, the results should encourage clinicians and lay health workers to be vigilant regarding the adverse effects of treatment, because they can seriously affect physical and emotional QoL.

## Background

By reducing the morbidity and mortality associated with HIV/AIDS, public-sector antiretroviral treatment (ART) has had a dramatic impact on the quality of life (QoL) of HIV/AIDS patients in industrialized countries [1-9]. These treatment regimens have also generated numerous socio-medical studies [1-4,6,10,11]. The South African Government has acknowledged the importance of QoL outcomes by stipulating in its Comprehensive Plan that 'improvements in patient well-being and quality of life' are the primary targets of the programme [12]. Although promising, the QoL outcomes for large-scale public ART programmes in developing countries are still preliminary [13-16]. As Ferradini et al. stated, studies of medium- to long-term cohorts of patients are still scarce and have usually been performed on very limited numbers of patients [17].

QoL is a multi-dimensional construct [18-20]. Quantitatively, it may be considered to have two dimensions: the physical and emotional [19-23]. In this context, the first measures the impact of disease on physical QoL by assessing the associated physical and functional limitations. The second addresses changes in the emotional states of patients, with an emphasis on mood, depression, and life satisfaction [24,25]. Compared with the basic physical outcomes of ART, far less is currently known about the ways in which and the extent to which ART affects the emotional QoL of patients, especially those living in resource-poor settings [4,26-29].

Previous studies by Louwagie et al. (2007) and Wouters et al. (2007) reported a strong positive association between the first six months of ART and the emotional and physical QoL of patients enrolled in South Africa's public-sector ART programme. The initial months of ART were associated with significant improvements in physical QoL [30,31]. Furthermore, patients receiving ART reported significantly higher levels of emotional well-being than patients awaiting treatment. These results indicate that ART is not only directly associated with emotional QoL, but is also indirectly associated with emotional QoL via the mediating variable physical QoL [30,31].

Although these studies have provided important insights into the impact of the South African public ART programme on the QoL of HIV/AIDS patients, they have been limited by the availability of only baseline data. Both studies stated that further longitudinal research was needed to clarify the link between ART and self-reported physical and emotional health outcomes over time. Furthermore, their cross-sectional designs compared patients awaiting ART with patients who had been receiving ART for less than six months. Although symptoms related to the adverse effects of treatment have been associated with lower QoL scores [17,32-35], a variable that measures adverse effects could not be included because patients awaiting treatment could not report such effects [30].

The first objective of this study was to overcome these study limitations and extend the current literature by examining the changes in physical and emotional QoL (differences between the baseline and the six-month follow-up scores) among patients enrolled in South Africa's public-sector ART programme. This longitudinal approach allowed us to address our second objective and include data on the adverse effects of treatment, because the literature shows that drug toxicity can have a substantial impact on patients' QoL [17,32-35].

## Methods

### Data collection

This study is part of an ongoing cohort study of patients enrolled in the public-sector ART programme in the Free State Province of South Africa. The research was approved by the Ethics Committee of the Faculty of Humanities of the University of the Free State.

The sampling frame consisted of a list of names obtained from the Provincial Department of Health of adult patients certified as medically ready to commence ART (CD4 < 200 cells/μL and/or WHO stage IV) within two months of the first patient having received his/her treatment. The list distinguished those patients who were receiving treatment at baseline ('treatment' patients) and those who were certified as ready to commence treatment but were not yet receiving it ('non-treatment' patients). Eighty patients were sampled randomly from this list for each of the five districts of the province (i.e., Lejweleputswa, Motheo, Thabo Mofutsanyana, Fezile Dabi, and Xhariep), proportional to the number of patients per clinic and per treatment group (treatment and non-treatment patients). Because there were less than 80 eligible patients in Xhariep, a census of all treatment and non-treatment patients was conducted.

A total of 371 study participants were recruited into the study (268 treated and 103 untreated patients). Only patients who were undergoing treatment at baseline (n = 268) were included in this study, because it assesses the impact of ART on the QoL of patients. This study used two waves of panel data to examine the impact of ART on QoL in the medium term. At baseline (< 6 months ART), trained enumerators conducted face-to-face interviews with 268 treatment patients using a standard questionnaire, after the written consent of all the patients had been obtained. Approximately six months later (< 12 months ART), 234 of the original cohort of patients were re-interviewed using an updated version of the questionnaire.

### Measures

**Figure 1:**
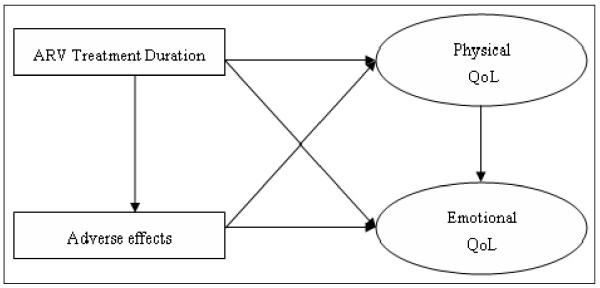
**The conceptual model, showing the hypothesized relationships between ART duration, adverse effects, and physical and emotional quality of life (QoL)**.

Figure 1 shows the conceptual model to be tested. The only exogenous variable is the length of treatment time. We measured the adverse effects of ART using a construct that categorized patients as having no adverse effects, mild adverse effects, or disruptive adverse effects. Incorporating the severity of the adverse effects is important because many adverse effects are related to mild toxicity, which can be treated easily. A wide variety of good measures of quality exist for use in HIV/AIDS populations when physical criteria are examined [29,36]. To assess physical QoL in our study, a latent variable was constructed using EuroQoL 5D – a standardized, widely validated and tested instrument developed by the EuroQoL Group to assess five generic aspects of current health (mobility, self-care, limitation of activities, pain, and mood). This instrument has shown good construct validity in AIDS trials in resource-poor settings [6,37-41]. Respondents could indicate one of three levels of impairment in each health domain: none, some/moderate, or extreme. The five items (and the entire questionnaire) were translated into Afrikaans, Xhosa, Zulu, and Sotho. The Afrikaans, Xhosa, and Zulu versions of the EQ-5D are recognised as official translations by the EuroQoL Group and have shown equivalent reliability and validity [30,40,42]. The Sotho instrument was translated for the purpose of this study and further research is required to ensure its measurement validity. The EQ-5D is a generic instrument developed to describe and evaluate overall health-related QoL. However, in this study, a distinction was drawn between the physical and emotional outcomes of ART. The "mood" item in the EQ-5D assesses an aspect of emotional health and was not included as an indicator of physical QoL. Therefore, a latent variable measuring the physical QoL of patients was constructed using only the first four dimensions of the EQ-5D.

Measures of overall emotional or psychological well-being are generally based on self-reports. This is called subjective well-being (SWB) [43-45]. SWB is an important indicator of QoL because it describes a person's multi-dimensional evaluation of his/her life, including affective evaluations of moods and emotions, and cognitive judgements of life satisfaction [45-49]. There is currently a consensus that SWB consists of three primary components: life satisfaction, pleasant affect, and low levels of unpleasant affect [44,47,48,50,51]. SWB has previously been used successfully to evaluate people's well-being in South Africa [30,43,49]. Patient responses to a standard five-point scaled item regarding overall life satisfaction were used to measure the cognitive part of SWB. An item asking about global happiness was used as a measure of positive emotions. The fifth EuroQoL item, measuring anxiety and depression, was used to measure the third component of SWB, the lack of unpleasant affect. This instrument has shown good reliability and construct validity in earlier studies of QoL in ART patients in South Africa [30].

### Data analysis

Structural equation modelling (SEM) was used to assess firstly, the influence of ART on physical and emotional QoL, and secondly, the impact of physical QoL on emotional QoL. This model also included the impact of adverse treatment effects on the patients' physical and emotional QoL. SEM is a combination of factor analysis and path analysis, and is particularly well suited to addressing longitudinal analytical problems. Unlike multiple regression models, SEM permits the simultaneous assessment of multiple dependent variables in a single model. SEM also allows the examination of both the direct and indirect effects of one variable on another. Furthermore, with SEM, a given construct can be treated as both an independent and a dependent variable, providing a model that more closely parallels the complex nature of most social and clinical phenomena (path analysis). Finally, SEM allows the examination of the relationships among both measured and latent variables (factor analysis), which often provides a more precise measure of complex social phenomena than do single measurements [52]. All data analysis was performed with the statistical software package LISREL Version 8.72.

## Results

### Sample description

The mean age of this sample of people living with HIV/AIDS was 37.9 years (SD = 8.6). Of the 268 ART patients included in this study, 66.8% were women. Overall, the patients described their physical QoL as good or excellent, with a relatively small improvement over time. The vast majority of patients had no problems with walking about (81.3% at baseline, 83.2% at follow-up), with washing or dressing themselves (94.0% at baseline, 94.8% at follow-up), or with performing their usual activities (80.6% at baseline, 85.8% at follow-up). Pain and discomfort were more common, and approximately four of every 10 respondents (39.6% at baseline, 37.5% at follow-up) reported at least some pain or discomfort. Generally, the respondents' emotional QoL was also high: 70.1% of respondents reported no anxiety or depression and 77.6% of respondents were fairly happy to very happy with their life in general, showing high levels of positive affect. The patients' life satisfaction was slightly lower, insofar as 44.0% of patients were dissatisfied to very dissatisfied with the way their life was going. At follow-up, patients emotional QoL was slightly better: 74.1% of respondents were neither anxious nor depressed, 72.4% were fairly happy to very happy, and only 38.8% were dissatisfied with their life in general (additional file 1 displays the descriptive statistics of the sample).

After six moths of ART, 55.1% of patients experienced some adverse effects of the antiretroviral medication. The most reported adverse effects were nausea, skin problems, and dizziness. When we measured the severity of these adverse effects, 58.9% of patients who reported adverse effects experienced them as very disruptive. Approximately six months later, drug substitution and symptomatic treatment had resulted in only 27.3% of patients reporting any adverse effects of treatment. However, 66.7% of these patients experienced these adverse effects as very disruptive. Between baseline and follow-up, 17.7% of the patients changed treatments. Of the patients evaluated, 56.0% started treatment with stavudine, lamivudine, and efavirenz, and 41.8% started treatment with stavudine, lamivudine, and nevirapine. Approximately six months later, 62.3% of patients were using the efavirenz-based regimen and only 36.4% were receiving nevirapine-based first-line ART. A recent clinical study by van Leth et al. (2005) indicated that both regimens are effective in patients with advanced HIV disease and that possible differences, if they exist, are small [53].

### Attrition analysis

The attrition rate of 13% (34 patients) between the two interviews was mainly caused by death (n = 15), refusal to be interviewed (n = 8), and our failure to determine the current whereabouts of the patient (n = 5). Attrition analysis of the study variables (physical and emotional QoL, adverse effects) revealed that responders had significantly better physical and emotional QoL at baseline than those of non-responders and reported fewer adverse effects than did non-responders.

### Measurement model

Because SEM is a hybrid of factor analysis and path analysis, a two-step approach is recommended. Separate assessments of the measurement and structural models prevent the good fit of one model compensating for (and potentially masking) the poor fit of the other (additional file 2 displays the correlations among all study variables). Table 1 shows the final measurement model, which has acceptable practical fit indices. As in our previous study, the composite reliability shows the excellent consistency of the indicators in measuring both latent variables: physical and emotional QoL. The discriminant validity of the constructs is supported by the χ^2 ^difference test and the variance extracted test. Combined, these findings support the reliability and validity of the two constructs and their indicators.

**Table 1:** Item analysis, goodness-of-fit, and reliability assessment of the measurement model (n = 268)

**Scale**	**Standardized Loadings^1^**	***t *Value**
**Physical QoL**		
Mobility	0.787***	13.883
Usual activities	0.936***	34.920
Pain	0.829***	20.995
Self-care	0.938***	23.281
**Emotional QoL**		
Satisfaction with life	0.730***	10.561
Pleasant affect	0.946***	11.394
Absence of unpleasant affect	0.798***	7.975

**Test for Fit**	**Measurement Model**	**Criteria for Good Fit**
RMSEA	0.0312	< 0.05
Normed Fit Index (NFI)	0.985	> 0.9
Non-Normed Fit Index (NNFI)	0.993	> 0.9
Comparative Fit Index (CFI)	0.996	> 0.9

**Reliability**		**Criteria for Reliability**
Composite reliability (Phys. QoL)	0.892	> 0.7
Composite reliability (Emo. QoL)	0.829	> 0.7

**Validity**		**Criteria for Validity**
χ^2 ^difference test	42.115 (1); *P *< 0,001	*P *< 0.05
Extracted variance test (Phys. QoL)	0.881	> 0.5
Extracted variance test (Emo. QoL)	0.812	> 0.5

*** *P *< 0.001

^1^The standardized factor loading is the standardized regression coefficient of the latent variable on the indicator (the measured variable). Squaring the factor loading gives us the percentage of variance of the measured variable explained by the latent variable.

### Structural model

Table 2 summarizes the parameter estimates of the structural model. The root mean square error of approximation (RMSEA) indicates the closeness of fit of the overall model, with reasonable errors of approximation in the population. Other goodness-of-fit statistics suggest that the structural model not only fits adequately, but also withstands the tests of parsimony (RMSEA = 0.0312, comparative fit index [CFI] = 0.996, parsimony normed fit index [PNFI] = 0.630).

**Table 2:** Standardized LISREL coefficients and summary of the structural model (n = 268)

**Path**	**Path Coefficient**	***t *Value**
ARV Treatment Duration → Adverse Effects	-0.38***	-6.031
Adverse Effects → Physical QoL	-0.27**	-2.729
Adverse Effects → Emotional QoL	-0.15*	-1.966
ARV Treatment Duration → Physical QoL	-0.03^a^	-0.329
ARV Treatment Duration → Emotional QoL	-0.08^a^	-0.761
Physical QoL → Emotional QoL	0.33**	2.971

**Test for Fit**	**Structural Model**	**Criteria for Good Fit**

RMSEA^1^	0.0312	< 0.05
Normed Fit Index (NFI)	0.985	> 0.90
Non-Normed Fit Index (NNFI)	0.993	> 0.90
Comparative Fit Index (CFI)	0.996	> 0.90
Parsimony Goodness of Fit Index (PGFI) ^2^	0.433	
Parsimony Normed Fit Index (PNFI) ^2^	0.630	

^a ^Not statistically significant. * *P *< 0.05, ** *P *< 0.01, *** *P *< 0.001

^1^RMSEA, root mean square error of approximation. RMSEA is a measure of the discrepancy per degree of freedom for the model and reflects badness-of-fit per degree of freedom. A value for RMSEA of 0.05 or less indicates a close fit of the model in relation to the degrees of freedom.

^2^These fit indices are relative fit indices that are adjustments of the goodness of fit index (GFI) and the normed fit index (NFI). The adjustments are made to penalize the models that are less parsimonious, so that simpler theoretical models are favoured over more complex ones. Although no threshold levels have been recommended for these indices, higher values are preferable.

### Physical and emotional QoL

The model tested the impact of ART on physical and emotional QoL during the second half of the first year of ART. The first part estimated the direct and indirect effects of treatment duration on both QoL measures. Firstly, the treatment variable had no significant impact on physical QoL, meaning that the additional six months of ART did not further improve the physical well-being of the patients. Secondly, patients did not report significantly higher emotional QoL at follow-up than that at baseline. However, the lack of a negative impact of treatment duration on QoL means that the improved QoL following the start of treatment was maintained at follow-up. Finally, there was a strong significant positive association between physical and emotional QoL. The patients' emotional QoL increased by 0.33 standard deviations when their physical QoL increased by one standard deviation (*P *< 0.001).

### Adverse effects

Unlike previous studies, this model included the adverse effects of treatment as a dependent variable on treatment duration and a predictor of physical and emotional QoL. Firstly, treatment duration was significantly associated with the experience of adverse effects (β = -0.38, *P *< 0.001). Patients at follow-up reported significantly fewer adverse effects than did patients at baseline. The amount of explained variance in the experience of adverse effects was 0.20. Secondly, the variable measuring adverse effects had a strong negative impact on physical QoL. The analysis shows that the respondent's physical QoL decreased by 0.27 standard deviations when the patient reported adverse effects (*P *< 0.01). The amount of explained variance in physical QoL was 0.15. Finally, these results indicate that adverse effects were also negatively associated with the emotional well-being of ART patients. The significant path coefficient of -0.15 implies that their emotional QoL increased by 0.15 standard deviations in the absence of adverse effects. The squared multiple correlation coefficient, R^2^, for the structural equation predicting emotional QoL was 0.40, indicating that 40% of the variation in emotional QoL can be explained by ART, the associated physical health benefits, and the absence of adverse effects.

## Discussion

This study assessed the physical and emotional QoL in a sample of 268 patients enrolled in South Africa's public-sector ART programme after six and 12 months. Overall, the physical and emotional QoL of the respondents were high and the improved physical and emotional QoL that were demonstrated in previous studies were sustained over the 12-month study period. Such favourable QoL outcomes are similar to those reported in industrialized societies [3,7,9,31,54-56]. This longitudinal study provides evidence that the South African public-sector ART programme is effective in delivering sustained improvements in patient well-being, confirming universal treatment access as a key policy priority.

This study also evaluated the impact of the adverse effects of medication on the patients' physical and emotional QoL. Adverse effects to ART significantly and negatively influenced patients' physical and emotional QoL. However, our longitudinal data showed that patients reported significantly fewer adverse effects at follow-up than at baseline. The most cited adverse effects (dizziness, nausea, and skin problems) are related to mild toxicity, which does not require the discontinuation of therapy or drug substitution (17.7% of patients changed treatments) and can be overcome by symptomatic treatment [57].

Despite favourable QoL outcomes, two considerations leave little room for complacency. The first one emerges when we link the lack of additional improvement in QoL to the reduction in adverse effects following early drug toxicity. In contradiction to the suggestion of Liu et al. (2006), the lack of additional long-term effects on QoL does not reflect a balance between reduced HIV-related symptoms and additional adverse effects from ART [35]. All other things being equal, a reduction in drug toxicity would be expected to result in a further improvement in QoL. Mannheimer et al. (2005) suggested that pill fatigue causes a decline in adherence levels, which is related to lower levels of physical and emotional QoL [3]. Further research is required to understand the cause of the lack of further QoL improvement when the adverse effects of ART decrease over time. Secondly, our results indicate a strong negative association between the adverse effects of ART and the physical and emotional QoL of patients. Although our results suggest a decline in adverse effects over time, other research has shown that many adverse effects of treatment only develop in the later stages of treatment (e.g., lipoatrophy) [57-61]. These results should encourage clinicians and lay health workers to be vigilant regarding the adverse effects of treatment, because they can seriously affect emotional and physical QoL.

### Strengths and limitations

The strengths of this study include its longitudinal character and the availability of information on an understudied population. However, it has some limitations. Because of the limited generalizability of the study findings, we can only ascribe the outcomes to patients enrolled in a public-sector ART programme and, more specifically, to patients enrolled in South Africa's Free State Province ART programme. Secondly, although the analysis focused on the impact of treatment duration and adverse effects on physical and emotional QoL, the structural equation model offers an incomplete explanation of the patients' QoL. Patient characteristics (age, sex) and socio-economic traits (education level and income) were tested as predictors of the patients' QoL but did not improve the model significantly. Other potentially relevant predictors of QoL (e.g., ART adherence, symptomatic interventions, and co-treatments) were not available in the data set.

## Conclusion

Although our knowledge of the complex relationships between ART and the multi-dimensional construct QoL in a resource-poor setting is still in its infancy, these analyses have both practical and theoretical implications. Theoretically, this study contributes to our understanding of the impact of ART on the different dimensions of QoL of patients enrolled in a public-sector ART programme in a resource-limited setting. Physical QoL and the adverse effects of treatment have a significant impact on emotional QoL, which draws greater attention to the emotional dimension of ART health outcomes. This research shows that emotional QoL correlates with physical health measures, but cannot be reduced to a mere component of physical QoL. Therefore, the general focus of research on physical outcomes is too narrow.

In practical terms, this study has shown that HIV patients enrolled in South Africa's public sector ART programme have similar QoL outcomes to those of patients in industrialized settings. The improvement in QoL was also sustained throughout the 12-month study period, indicating the medium-term efficacy of a public-sector ART programme in a high-prevalence, resource-limited setting. However, vigilance is needed because our findings show that the adverse effects of treatment can significantly reduce the chance of a successful ART scale-up. Further research is required to inform policy-makers of how to maximize the benefits of ART in the context of a massive epidemic and an often overstretched health system.

## Competing interests

The authors declare that they have no competing interests.

## Authors' contributions

EW participated in the design of the study, performed the statistical analysis, and wrote the manuscript. DvR supervised the overall management of the longitudinal study and gave advice in interpreting the results. DvR, CH, and HM were involved in revising the article for important intellectual content. All authors read and approved the final manuscript.

## Pre-publication history

The pre-publication history for this paper can be accessed here:



## Supplementary Material

Additional file 1 **Descriptive analysis of the adverse effects of treatment and the physical and emotional quality of life (QoL) in a sample of ART patients (n = 268).** Click here for file

Additional file 2 **Correlations among all variable.** Click here for file
