# Supplementary material for: Physical and emotional health outcomes after 12 months of public-sector antiretroviral treatment in the Free State Province of South Africa: a longitudinal study using structural equation modelling
Source: BMC Public Health. 2009 Apr 15;9:103. doi: 10.1186/1471-2458-9-103 (PMC2678117; doi:10.1186/1471-2458-9-103)
Supplement: Additional file 1 — Descriptive analysis of the adverse effects of treatment and the physical and emotional quality of life (QoL) in a sample of ART patients (n = 268). [file 1471-2458-9-103-S1.doc]

**Additional file 1. Descriptive analysis of the adverse effects of treatment and the physical and emotional quality of life (QoL) in a sample of ART patients (n = 268)**

|  | **Baseline** | | | | | | **Follow-up** | | | | | |
| --- | --- | --- | --- | --- | --- | --- | --- | --- | --- | --- | --- | --- |
|  | **Total** | **Age (years)** | | | **Gender** | | **Total** | **Age (years)** | | | **Gender** | |
|  |  | 0-30 | 30-50 | 45+ | Male | Female |  | 0-30 | 30-50 | 45+ | Male | Female |
| **Adverse effects of treatment** |  |  |  |  |  |  |  |  |  |  |  |  |
| No adverse effects, % | 44.9 | 50.0 | 46.2 | 38.5 | 41.8 | 46.6 | 72.7 | 77.5 | 71.1 | 72.7 | 76.9 | 70.6 |
| Mild adverse effects, % | 20.0 | 17.5 | 21.1 | 19.2 | 23.3 | 19.0 | 9.1 | 10.0 | 7.4 | 12.7 | 9.0 | 9.1 |
| Disruptive adverse effects, % | 35.1 | 32.5 | 32.7 | 42.3 | 34.9 | 34.4 | 18.2 | 12.5 | 21.5 | 14.6 | 14.1 | 20.3 |
| **Quality of Life** |  |  |  |  |  |  |  |  |  |  |  |  |
| Physical QoL |  |  |  |  |  |  |  |  |  |  |  |  |
| Mobility, mean (SD)¹ | 1.80 (0.41) | 1.85 (0.36) | 1.82 (0.42 | 1.75 (0.44) | 1.74 (0.49) | 1.84 (0.37) | 1.83 (0.39) | 1.85 (0.36) | 1.83 (0.39) | 1.78 (0.42) | 1.85 (0.36) | 1.82 (0.40) |
| Self-care, mean (SD)¹ | 1.92 (0.30) | 1.89 (0.43) | 1.95 (0.25) | 1.90 (0.30 | 1.87 (0.40) | 1.96 (0.22) | 1.95 (0.22) | 1.97 (0.16) | 1.93 (0.25) | 1.96 (0.19) | 1.96 (0.19) | 1.94 (0.24) |
| Usual activities, mean (SD)¹ | 1.77 (0.49) | 1.76 (0.52) | 1.76 (0.51) | 1.82 (0.43) | 1.71 (0.58) | 1.81 (0.44) | 1.84 (0.40) | 1.88 (0.40) | 1.84 (0.41) | 1.84 (0.37) | 1.86 (0.39) | 1.84 (0.40) |
| Pain/discomfort, mean (SD)¹ | 1.55 (0.59) | 1.55 (0.69) | 1.53 (0.58) | 1.60 (0.56) | 1.55 (0.60) | 1.55 (0.59) | 1.59 (0.57) | 1.66 (0.57) | 1.56 (0.59) | 1.61 (0.49) | 1.56 (0.57) | 1.60 (0.57) |
| Emotional QoL |  |  |  |  |  |  |  |  |  |  |  |  |
| Life satisfaction, mean (SD)² | 2.36 (1.20) | 2.47 (0.95) | 2.26 (1.26) | 2.50 (1.21) | 2.36 (1.25) | 2.36 (1.18) | 2.39 (1.14) | 2.51 (1.08) | 2.30 (1.21) | 2.49 (1.02) | 2.44 (1.13) | 2.36 (1.15) |
| Positive affect, mean (SD)² | 3.02 (0.96) | 2.96 (0.86) | 3.01 (1.04) | 3.10 (0.80) | 3.01 (0.89) | 3.00 (0.99) | 2.87 (1.02) | 2.85 (1.09) | 2.91 (0.98) | 2.78 (1.08) | 2.82 (1.03) | 2.89 (1.02) |
| Negative affect, mean (SD)¹ | 1.63 (0.61) | 1.60 (0.61) | 1.62 (0.63) | 1.67 (0.57) | 1.55 (0.67) | 1.67 (0.58) | 1.71 (0.52) | 1.78 (0.42) | 1.70 (0.55) | 1.67 (0.51) | 1.69 (0.54) | 1.70 (0.50) |

¹ Minimum = 0 (severe problems), maximum = 2 (no problems)

² Minimum = 0 (very unsatisfied), maximum = 4 (very satisfied)
