# Supplementary material for: Physical and emotional health outcomes after 12 months of public-sector antiretroviral treatment in the Free State Province of South Africa: a longitudinal study using structural equation modelling
Source: BMC Public Health. 2009 Apr 15;9:103. doi: 10.1186/1471-2458-9-103 (PMC2678117; doi:10.1186/1471-2458-9-103)
Supplement: Additional file 2 — Correlations among all variable. [file 1471-2458-9-103-S2.doc]

**Additional file 2.** Correlations among all variables

| Lack of unpleasant affect |  |  |  |  |  |  |  |  | 1 |
| --- | --- | --- | --- | --- | --- | --- | --- | --- | --- |
| Pleasant affect |  |  |  |  |  |  |  | 1 | 0.746 |
| Life satisfaction |  |  |  |  |  |  | 1 | 0.674 | 0.657 |
| Self-care |  |  |  |  |  | 1 | 0.163 | 0.311 | 0.403 |
| Pain |  |  |  |  | 1 | 0.776 | 0.198 | 0.347 | 0.328 |
| Usual activities |  |  |  | 1 | 0.757 | 0.888 | 0.131 | 0.448 | 0.330 |
| Mobility |  |  | 1 | 0.713 | 0.700 | 0.740 | 0.068 | 0.258 | 0.435 |
| Adverse effects |  | 1 | –0.260 | –0.297 | –0.169 | –0.224 | –0.143 | –0.261 | –0.019 |
| ARV treatment duration | 1 | –0.420 | 0.065 | 0.080 | 0.037 | 0.071 | 0.143 | -0.074 | 0.079 |
|  | ARV treatment duration | Adverse effects | Mobility | Usual activities | Pain | Self-care | Life satisfaction | Pleasant affect | Lack of unpleasant affect |
